# Viral Reservoirs in Lymph Nodes of FIV-Infected Progressor and Long-Term Non-Progressor Cats during the Asymptomatic Phase

**DOI:** 10.1371/journal.pone.0146285

**Published:** 2016-01-07

**Authors:** C. D. Eckstrand, C. Hillman, A. L. Smith, E. E. Sparger, B. G. Murphy

**Affiliations:** 1 Department of Pathology, Microbiology, and Immunology, School of Veterinary Medicine, University of California, Davis, California, United States of America; 2 Veterinary Medical Teaching Hospital, School of Veterinary Medicine, University of California, Davis, California, United States of America; 3 Department of Medicine and Epidemiology, School of Veterinary Medicine, University of California, Davis, California, United States of America; CEA, FRANCE

## Abstract

**Background:**

Examination of a cohort of cats experimentally infected with feline immunodeficiency virus (FIV) for 5.75 years revealed detectable proviral DNA in peripheral blood mononuclear cells (PBMCs) harvested during the asymptomatic phase, undetectable plasma viral RNA (FIV *gag)*, and rarely detectable cell-associated viral RNA. Despite apparent viral latency in peripheral CD4+ T cells, circulating CD4+ T cell numbers progressively declined in progressor animals. The aim of this study was to explore this dichotomy of peripheral blood viral latency in the face of progressive immunopathology. The viral replication status, cellular immunophenotypes, and histopathologic features were compared between popliteal lymph nodes (PLNs) and peripheral blood. Also, we identified and further characterized one of the FIV-infected cats identified as a long-term non-progressor (LTNP).

**Results:**

PLN-derived leukocytes from FIV-infected cats during the chronic asymptomatic phase demonstrated active viral *gag* transcription and FIV protein translation as determined by real-time RT-PCR, Western blot and *in situ* immunohistochemistry, whereas viral RNA in blood leukocytes was either undetectable or intermittently detectable and viral protein was not detected. Active transcription of viral RNA was detectable in PLN-derived CD4+ and CD21+ leukocytes. Replication competent provirus was reactivated *ex vivo* from PLN-derived leukocytes from three of four FIV-infected cats. Progressor cats showed a persistent and dramatically decreased proportion and absolute count of CD4+ T cells in blood, and a decreased proportion of CD4+ T cells in PLNs. A single long-term non-progressor (LTNP) cat persistently demonstrated an absolute peripheral blood CD4+ T cell count indistinguishable from uninfected animals, a lower proviral load in unfractionated blood and PLN leukocytes, and very low amounts of viral RNA in the PLN.

**Conclusion:**

Collectively our data indicates that PLNs harbor important reservoirs of ongoing viral replication during the asymptomatic phase of infection, in spite of undetectable viral activity in peripheral blood. A thorough understanding of tissue-based lentiviral reservoirs is fundamental to medical interventions to eliminate virus or prolong the asymptomatic phase of FIV infection.

## Introduction

Feline immunodeficiency virus (FIV) is a naturally occurring lentivirus that infects domestic cats and is associated with life-long viral persistence and progressive immunopathology. The disease is characterized by three distinct and sequential stages including an acute viremic stage, a prolonged and variable asymptomatic phase, and a terminal acquired immunodeficiency disease stage [1]. The host-viral interactions in the acute and early asymptomatic stages of FIV infection have been studied extensively. During the acute stage of infection there is wide viral dissemination to many cell and tissue types, a high plasma viral load, a decrease in CD4+ T cells and an inverted CD4:CD8 ratio in the peripheral blood [2–6]. FIV has a relatively diverse cellular tropism due to the presence of the viral receptor (CD134) on many leukocyte subsets [7]. The acute phase of infection is followed by a prolonged asymptomatic phase, in which the cat remains clinically healthy despite a progressive decline in the peripheral blood CD4+ T cell numbers and leukocyte function [8,9]. Possibly as a result of the prolonged expense of maintaining experimentally infected animals, the chronic asymptomatic phase and the events associated with the transition into the terminal acquired immunodeficiency stage are poorly described and remain under-investigated.

Our laboratory has closely followed a cohort of experimentally FIV-infected cats for approximately 5.75 years. These cats are currently in the chronic asymptomatic phase of infection. Observations of this cohort of infected animals have revealed that viral *gag* RNA (here-on abbreviated as viral RNA or vRNA) is generally undetectable in peripheral blood mononuclear cells (PBMCs) and is undetectable in plasma suggestive of an inactive viral transcription status in the peripheral blood [10,11]. Our group has demonstrated that within this FIV-infected experimental cohort, viral latency in peripheral blood-derived CD4+ T cells is associated with epigenetic modification of histone proteins physically associated with the FIV 5’ LTR (viral promoter) [11,12]. Despite an inactive viral transcription status and a condensed chromatin pattern of the FIV LTR, circulating CD4+ T cell numbers have progressively declined over time in this cohort of cats [13].

Many details concerning the pathogenesis of the chronic asymptomatic phase of FIV infection remain poorly characterized, and thus we sought to further define the anatomic and cellular distribution of viral persistence, viral replication status, and the immunopathologic profile during this phase. The overall goal of these studies was to investigate the discordance between an inactive viral activity status and a progressive immunopathology in the peripheral blood. We addressed this goal with the hypothesis that viral persistence is associated with active viral replication within lymphoid tissues in FIV-infected cats during the asymptomatic phase. This experimental cohort also provided a detailed description of the virological and immunopathological characteristics associated with a FIV-infected LTNP cat.

## Materials and Methods

### Animals, serial peripheral blood assays, and PLN procurement

All experimental study protocols were approved by the University of California Davis Institutional Animal Care and Use Committee (IACUC, permit #18155). Surgical anesthesia and post-operative pain management protocols were also IACUC-approved (permit number above) and employed combinatorial injectable, inhalational and transmucosal pharmaceuticals. Six specific pathogen free (SPF) kittens were purchased from the breeding colony of the Feline Nutrition and Pet Care Center, University of California, Davis (UC Davis). At the time of purchase, kittens ranged in age from 4 to 5 months and were housed in the Feline Research Laboratory of the Center for Companion Animal Health, UC Davis. Four kittens were intramuscularly inoculated with FIV-C subtype (Paddy-Gammer strain) viral inoculums [14] (kittens 165, 184, 186 and 187) and two control kittens (183 and 185) were mock-inoculated intramuscularly with 1 ml of sterile culture media and monitored as previously described [15]. The FIV-C biological isolate was provided by Drs. E. Hoover (Colorado State University) and N. Pedersen (UC Davis). The study cats were housed in cat groups (4 cats FIV positive and 2 cats FIV negative) and maintained in individual rooms that were cleaned daily. The rooms had multiple perches and a variety of cat toys and boxes for enrichment, play and exercise. The cats were fed, observed and litter boxes changed on a daily basis. Room lighting cycles were seasonally adjusted.

Peripheral blood samples were collected once to twice per month over the 5.75 year study period and analyzed for plasma vRNA, PBMC-associated viral *gag* DNA (provirus) and vRNA, and absolute CD4+ T cell counts. The absolute CD4+ T cell count in blood was derived from the absolute white blood cell count and subset frequency determined by flow cytometry. Between 274 and 290 weeks post-infection, bilateral popliteal lymph nodes (PLNs) were surgically removed from each cat under general anesthesia using a sterile technique approved by the UC Davis IACUC Committee. At the time of surgery, 10 mls of peripheral blood was also collected from each animal. The surgical pharmaceutical protocol included a subcutaneous premedication injection of atropine (0.02mg/kg) and butorphanol (0.3mg/kg), intravenous induction with ketamine (5mg/kg) and midazolam 0.5mg/kg, inhalational anesthesia maintenance with oxygen and 2% isoflurane via an endotracheal tube, and intraoperative intravenous ampicillin (20mg/kg). Post-operative medications consisted of buprenorphine (0.02mg/kg) administered transmucosally twice daily for 3–7 days as needed, and amoxicillin trihydrate/clavulanate potassium given orally twice daily (6.25mg/lb) for 10 days.

### Peripheral blood and tissue leukocyte collection and flow cytometry

PBMCs were harvested from whole blood by Ficoll-Hypaque density gradient centrifugation (Sigma, St. Louis, Mo.). PLNs were surgically harvested and the weight of each node was recorded after the aseptic removal of pericapsular adipose tissue. For each cat, approximately one third of a single node was fixed in 10% buffered formalin for histologic and immunohistochemical studies. Remaining tissue was dissociated by manual disruption and processed for individualized leukocytes by filtration through a 70um mesh sieve (BD Falcon). Isolated PBMCs and PLN leukocytes were resuspended and enumerated using both an automated cell counter (Coulter ACT diff, Beckman Coulter) and a manual hemocytometer. Approximately 7 x 10^6^ PBMCs and PLN leukocytes were assayed for cellular phenotype by flow cytometry and the remainder were viably frozen in fetal bovine serum with 10% DMSO and stored in liquid nitrogen. The antibodies used for flow cytometry included anti-feline CD4 (clone FE1.7B12), anti-feline CD8 (clone FE1.10E9), anti-canine CD21 (clone CA2.1D6), anti-canine 11b (clone CA16.3E10), anti-feline MHC II (clone 42.3), and anti-human CD14 (Biorad, clone Tuk4). All of the antibodies were obtained from Dr. Peter Moore (UC Davis) with the exception of anti-human CD14 (Biorad, clone Tuk4), previously reported to be a useful monocyte marker in cats [16]. The proportion of cells expressing a specific marker was determined by flow cytometry, using indirect immunofluorescence with a secondary fluorescein isothiocyanate (FITC)-conjugated horse anti-mouse IgG (Vector Laboratories Inc.) as previously described [15]. After staining, leukocyte phenotype proportions were determined using a Cytomics FC500 flow cytometer (Beckman Coulter), and FlowJo v8.6.3 software (Treestar). Positive populations were expressed as frequency of positive events over 200,000 total events.

### Leukocyte subset enrichment and *ex vivo* viral reactivation

Starting with freshly isolated PBMCs and PLN leukocytes (~10^7^ cells), CD4 and CD21 subsets were enriched by positive immunomagnetic selection using the primary antibodies described above, MS immunomagnetic columns (MACS Separation Columns, Miltenyi Biotec Inc.) and goat-anti mouse IgG-microbeads (Miltenyi Biotec) as previously described [15]. The purity of CD4 and CD21 immunomagnetic sorted cell populations was determined to be greater than 95% by flow cytometry. Enriched leukocyte subsets were subsequently interrogated for cell-associated viral DNA (provirus) and vRNA as described below.

The *ex vivo* reactivation assay was performed by co-culturing previously cryopreserved PLN-derived leukocytes (10^7^) with allogeneic SPF PBMCs (2.5 x 10^6)^ in RPMI media supplemented 5μg/ml mitogen concanavalin-A (Con-A; ThermoFisher Scientific) and human IL-2 (NIH AIDS Reagent Program) as previously described [15]. Con-A was removed by refreshing the culture media after 24 hours. On days 7 and 14, the culture media was refreshed and aliquots (2 x 10^6^) of Con-A activated allogeneic SPF feline PBMCs were added. Clarified cell-free supernatants collected on days 7, 14, and 21, frozen at -80°C and later, were independently passaged onto approximately 7 x 10^6^ Con-A activated allogeneic SPF PBMCs. Cultured allogeneic cells were subsequently assayed for the presence of *gag* DNA (provirus) by real-time PCR 7 days after the addition of the clarified supernatant. Detectable *gag* provirus was interpreted to be consistent with successful *ex vivo* viral reactivation. This assay was performed on cryopreserved PLN leukocytes from all four FIV positive cats, and one negative control cat (185).

### Establishing the viral infection status of peripheral blood and PLN leukocytes

Viral RNA was isolated from clarified feline plasma and cell-associated RNA and DNA were co-isolated using commercially available kits (QIAmp Viral RNA Minikit and AllPrep DNA/RNA Mini Kit, Qiagen) according to manufacturer's instructions. Plasma vRNA and cell-associated RNA were DNase treated (Turbo DNase, Ambion) and reverse transcribed using the First-Strand cDNA Synthesis System for Quantitative RT-PCR (OriGene). Real-time PCR was performed in triplicate on an Applied Biosystems 7300 Real-Time PCR System with FIV_QT gag_ primers under conditions previously described [15]. A negative control reaction excluding reverse transcriptase was included for each set of cDNA samples. This real-time PCR assay has a detection limit of approximately 10 copies of FIV *gag* cDNA (data not shown) or 10^3^ copies of FIV *gag* cDNA per ml of feline plasma. Quantification of plasma vRNA copy number was based on a standard curve previously described [17]. For assay of cell-associated vDNA and vRNA, real-time PCR assays of feline *GAPDH* were included in parallel using the same nucleic acid samples to normalize input nucleic acid concentration.

### Viral protein detection by Western blot and immunohistochemistry

Cell lysates of unfractionated PBMC and PLN-derived leukocytes from FIV-infected and uninfected cats were prepared using a 0.25% deoxycholate and 0.1% sodium dodecyl sulfate (SDS) lysis buffer and sonication. Eight μg of cell lysate (determined by BCA protein assay kit; Thermo Scientific, Pierce) were loaded into a 4–20% SDS Precast Polyarcrylamide Gels (Expedeon) and separated by electrophoresis. Proteins were transferred to a PVDF membrane (Bio-Rad) overnight and then probed with monoclonal anti-Gag antibody (clone 43-1B9, N. Pedersen, UC Davis) at a 1:1000 dilution. Membranes were washed, followed by a 1-hour incubation with HRP conjugated-goat anti-mouse IgG antibody (Santa Cruz Biotechnology, 1:1000 dilution), and incubated for 1 minute with Pierce ECL Western Blotting Substrate (Thermo Scientific). Digital images were obtained via a FlourChem E imaging system (ProteinSimple).

Immunohistochemistry was performed on four-micron thick, formalin-fixed, paraffin-embedded lymph node sections, mounted on positively-charged slides, and air-dried overnight at 37°C. Sections were deparaffinized through xylene to reagent alcohol, and treated with 0.3% hydrogen peroxide in methanol for 30 minutes. Sections were then rehydrated to water through graded alcohols, stabilized in 0.1M phosphate buffered saline, pH 7.4 (PBS), and were exposed to citrate buffer, pH 6 (S1699 Dako) for 10 minutes at 95°C, and cooled for 20 minutes at room temperature. Sections were rinsed in PBS and treated with 10% normal horse serum for 20 minutes. Plasma from a serologically FIV positive domestic cat diluted 100-fold in PBS-Tween 20 (0.02%) was applied for 1 hour. This and all subsequent reagent incubations were performed at room temperature, and PBS-Tween 20 rinses were performed twice between reagent applications. An anti-feline horseradish peroxidase conjugated antibody (Cappel) diluted 400-fold was applied for 20 minutes to label bound FIV antibodies. Labels were visualized with NovaRed for peroxidase (SK-4800, Vector Laboratories). Sections were counterstained in Gill’s Hematoxylin, air-dried and sealed with a coverslip. Non-specific background was evaluated with a duplicate section receiving PBS in place of the primary antibody (negative control).

### Histology, immunohistochemistry, and image analysis

Immunohistochemistry (IHC) was utilized to phenotype PLN leukocytes using antibodies for feline CD3 (Peter Moore, UC Davis, clone CD3-12), CD79 (AbD Serotec, clone HM57), CD204 (Trans Genic, clone SRA-E5), and Ki67 (Dako, clone MIB-1) on 4 micron serial sections with a streptavidin biotin detection system (Biocare Medical) and following the protocol described above, but using an anti-mouse secondary antibody. Substituting a matched mouse IgG for the primary antibody served as the negative control. The proportion of CD3+ T cells within each lymphoid follicle and Ki67+ cells in the paracortical zones were quantified utilizing ImageJ software (National Institutes of Health). Briefly, photomicrographs of lymphoid follicles and paracortical zones (five different follicles/cat and three random paracortical regions/cat) were obtained. Using ImageJ, the image threshold was adjusted to black (IHC positive cells) and white to minimize non-specific background. IHC positive cells were quantified by specifying the lower limit of cell size (100 pixels) and accounting for overlapping cells. The image was then assessed for total number of nuclei using the image-based tool for counting nuclei (ITCN). The frequency of IHC positive cells was expressed as a division of the number of positive cells divided by total number of nuclei.

### Statistics

Graphical numerical data is presented as the mean of three or more values with the standard deviation or range represented by error bars. Statistical differences were determined by unpaired Student’s t-tests. A p value < 0.05 was considered to be statistically significant. Arcsine transformation was performed for all collected percentage data prior to statistical evaluation. Statistics were performed with Prism 6 software (GraphPad Software, Inc., La Jolla, CA).

## Results

### Peripheral immunopathology

**Fig 1:**
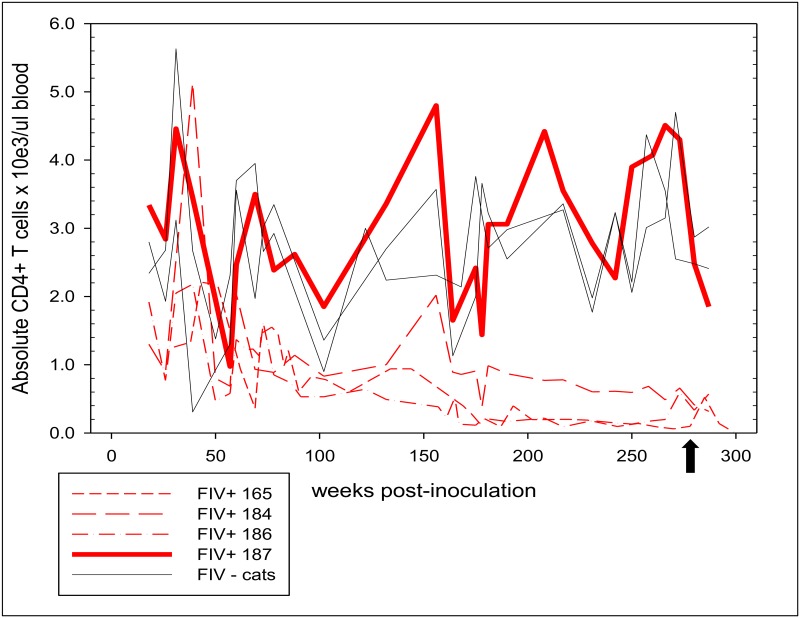
Progression of absolute CD4+ T cell counts in peripheral blood of FIV-infected and uninfected cats over 290 weeks post-inoculation. Systematic monitoring of the absolute CD4+ T cell counts in the peripheral blood of FIV-infected cats (dashed red lines) demonstrated a progressive decline below that of the two FIV-uninfected cats (gray lines), with the exception of cat 187 (bold red line, FIV-infected LTNP cat) who maintained an absolute CD4 T cell count indistinguishable from FIV-uninfected cats. The black arrow denotes the time of PLN removal.

Through flow cytometry and total WBC counts, absolute numbers of peripheral blood CD4+ T cells were serially determined for FIV-infected cats and compared to uninfected cats approximately once to twice a month for the duration of the study period. Three FIV-infected cats had absolute CD4+ T cell counts that declined over time (termed progressor cats) whereas uninfected cats generally had absolute counts >2000 cells/μl (Fig 1). The mean absolute circulating CD4+ T cell count of FIV-infected progressor cats from 0–290 weeks post infection was 774.5 cells/μl ± 709.7, while the mean for FIV-uninfected cats was 2714 cells/μl ± 965.2. A statistically significant difference was detected (p<0.0001) in absolute CD4+ T cell counts between FIV-infected progressor animals and uninfected cats over the entire study period. The progressor cats demonstrated very low absolute CD4+ T cell counts from 180 weeks post-infection onward with a mean of 364.8 cells/μl ± 260. Cats 165 and 186 demonstrated extremely reduced circulating CD4+ absolute counts at <200 cells/μl onward from weeks 202 and 167 post-infection respectively and nadirs of 60 and 90 cells/μl. FIV-infected cat 187 maintained an absolute CD4+ T cell count indistinguishable from the uninfected control animals throughout the 5.75-year monitoring period (mean = 3051 cells/μl ± 1175) and was thus defined as a FIV-infected LTNP.

### PLN weights and leukocyte subset proportions by flow cytometry

The mean weight of the FIV-infected cats' lymph nodes (210mg ± 62.6, n = 8) was significantly greater when compared to FIV-negative cat lymph nodes (123mg ± 45.7 n = 4, p = 0.0245) due to marked lymphoid hyperplasia in the FIV-infected cats' lymph nodes as determined by histology.

**Fig 2:**
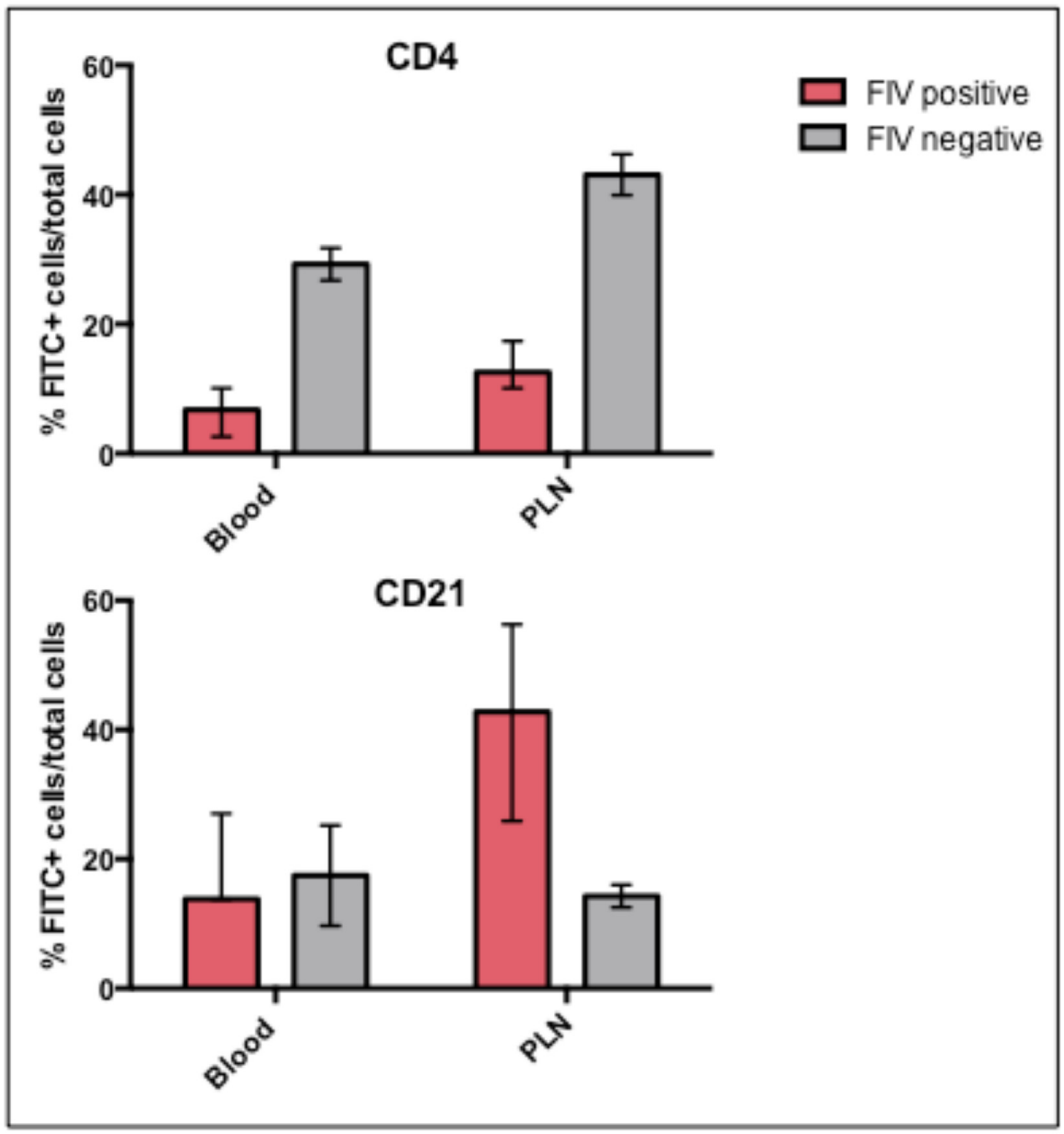
Percentages of CD4+ T cells and CD21+ leukocyte cell populations in the blood and PLNs of FIV-infected progressor and uninfected cats. Bars represent the mean, and the range by error bars.

Comparison of the different leukocyte subsets by flow cytometry analysis revealed a much lower frequency of CD4+ T cells in both the peripheral blood (as previously reported) and PLNs and a greater frequency of CD21+ B cells in the PLNs of FIV-infected progressor cats relative to uninfected cats (Fig 2). Statistical tests to determine significance were not performed due to insufficient numbers of animals. There was no notable differences in the proportions of CD8+, CD14+, CD11b+, or MHCII+ populations in the blood or PLN between FIV-infected progressor and uninfected cats (data not shown). The FIV-infected LTNP cat exhibited CD4+ and CD21+ leukocyte frequencies in both the peripheral blood and PLN indistinguishable from the FIV-uninfected cats (data not shown).

### *Ex vivo* viral reactivation

The presence of replication competent infectious virus from *ex vivo* reactivated PLN-derived leukocytes was detected in three of the four FIV-infected cats (Table 1). Clarified cell-free culture supernatants collected from primary PLN unfractionated leukocytes cultures were passaged onto SPF FIV-negative feline PMBCs at days 7, 14, and 21. After 7 days of the secondary culture, real-time PCR was performed to detect FIV-*gag* provirus in SPF PBMC DNA. A positive PCR result indicated that the clarified supernatant contained reactivated infectious virus. Infectious virus was detected in culture supernatant at day 7 for cat 165, day 14 for cat 186, and (interestingly) day 21 for the non-progressor cat 187. Integrated FIV provirus was not detected at any time point from FIV-uninfected cat (185, negative control) or FIV-infected cat 184 PLN-derived leukocytes.

**Table 1:** Detection of cell-associated FIV proviral DNA and viral RNA (*gag*) in temporally paired unfractionated blood-derived PBMCs and PLN-derived leukocytes. Proviral DNA was detected in unfractionated blood-derived PBMCs and PLN-derived leukocytes from all FIV-infected cats by real-time PCR. Viral RNA (FIV *gag*) was only detected in PLN-derived unfractionated leukocytes and was not detected (N.D.) in blood-derived leukocytes by real-time RT-PCR. Data are derived from triplicate real-time PCR reactions performed with cellular DNA or cDNA (FIV *gag* RNA).

	FIV DNA (provirus) copies FIV *gag* per 1 x 10^5^ cells		FIV RNA copies FIV *gag* per 10^6^ GAPDH		FIV propagated Days post reactivation
Cat #	Blood	PLN	Blood	PLN	PLN
165	+	+	N.D.	+	+
	7.5 x 10^2^	2.3 x 10^3^		5.23 x 10^5^	7
184	+	+	N.D.	+	N.D.
	1.05 x 10^3^	6.74 x 10^2^		6.96 x 10^3^	
186	+	+	N.D.	+	+
	4.17 x 10^2^	3.03 x 10^2^		4.96 x 10^4^	14
187	+	+	N.D.	+	+
	2.52 x 10^2^	1.51 x 10^2^		2.79 x 10^4^	21

### Plasma virus, proviral load and viral RNA quantification

**Fig 3:**
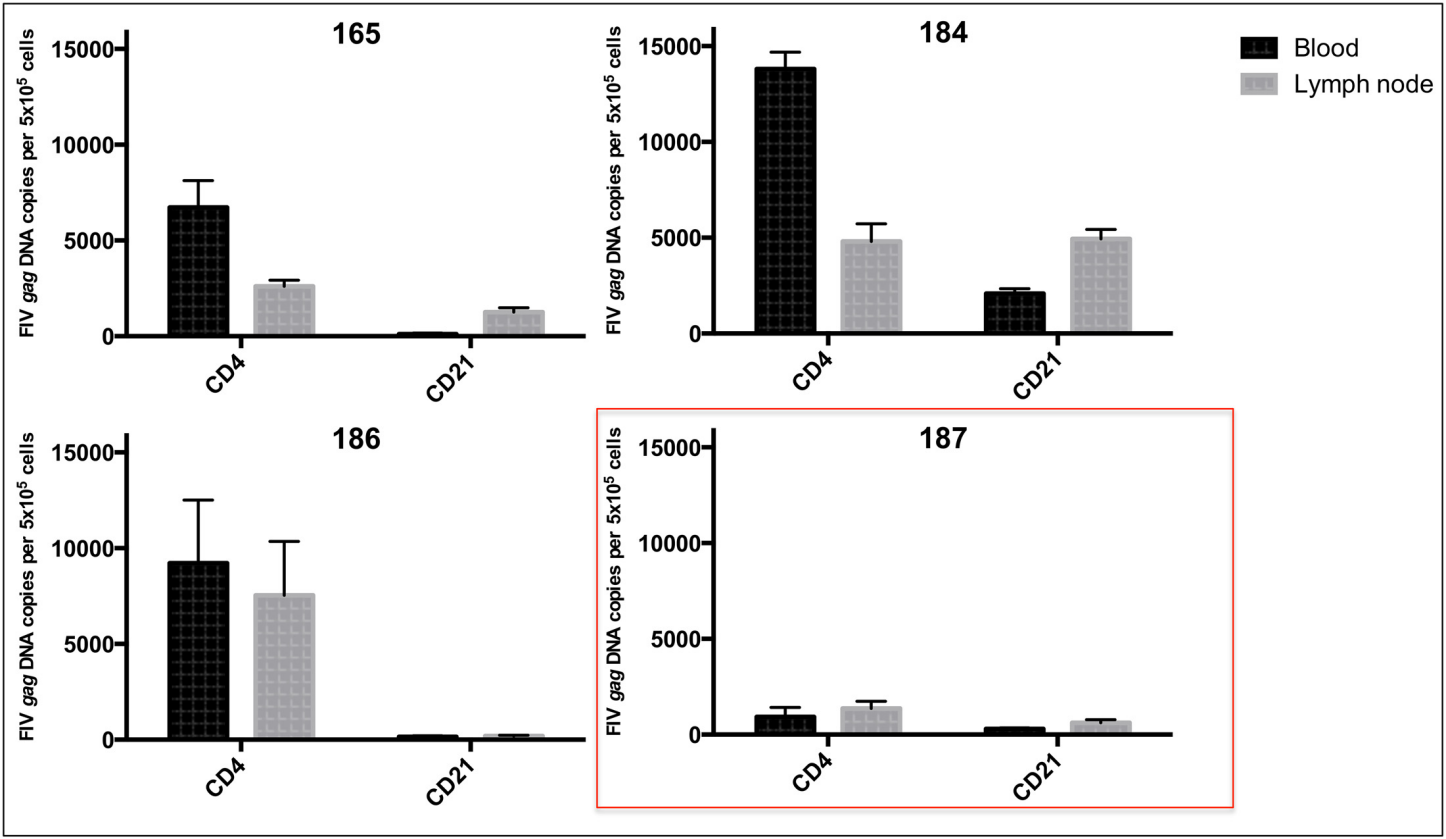
Proviral DNA load in CD4+ T cells and CD21+ leukocytes cells derived from the blood and PLN of each FIV-infected cat. The mean proviral load of blood and PLN-derived CD4+ T cells from LTNP cat 187 are less than those of progressor cats 165, 184 and 186.

During this study period, plasma vRNA was not detected within the limits of the real-time PCR assay for any of the FIV-infected and uninfected cats, consistent with our previous observations [10,11,18]. Proviral DNA loads for unfractionated PBMCs and PLN-derived leukocytes, as well as from blood and PLN enriched CD4+ and CD21+ populations were determined by real-time PCR. The mean proviral loads of unfractionated PBMCs and PLN leukocytes from progressor cats were 7.39 ± 3.16 x 10^2^ and 10.8 ± 10.6 x 10^2^ copies/ 5 x 10^6^ cells respectively, with no significant difference between blood and tissue compartments. With respect to enriched CD4+ T cells, a greater proviral load in PBMC-derived cells relative to PLN-derived cells was observed for progressor cats 165 and 184, but not 186 (Fig 3). Interestingly, the LTNP cat (187) showed a notably lower proviral load compared to progressor animals in unfractionated PBMCs, unfractionated PLN leukocytes (Table 1), and CD4+ T cells from both compartments (Fig 3).

**Fig 4:**
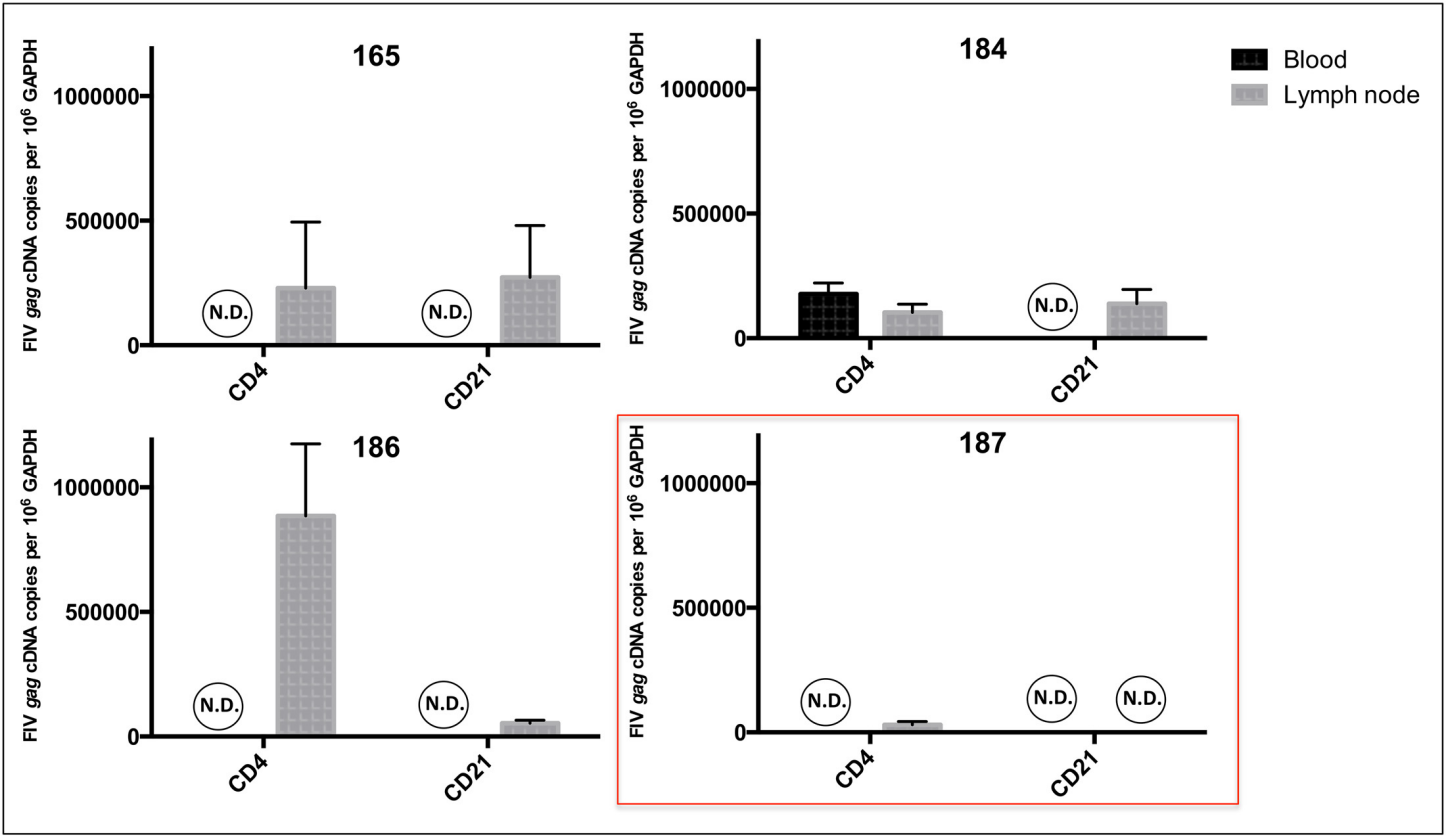
Viral *gag* RNA quantification in blood and PLN-derived CD4+ and CD21+ leukocyte subsets. Viral RNA was detected in PLN-derived CD4+ T cells and CD21+ cells of all FIV-infected progressor cats, while it was not detected in all of the blood-derived cells with the exception of cat 184 who had detectable vRNA in peripheral CD4+ T cells. In the non-progressor cat (187) vRNA was only detected in PLN-derived CD4+ T cells (red box).

Cell-associated vRNA was quantified by real-time PCR in both unfractionated PBMCs and PLN-derived leukocytes to test the hypothesis that active viral transcription persists in lymph node tissue in spite of persistently undetectable plasma vRNA and absent to rarely detectable cell-associated vRNA in PBMCs during the asymptomatic phase of infection. This hypothesis was supported by the detection of vRNA in unfractionated PLN-derived leukocytes of all four of the FIV-infected cats, and the absence of detectable vRNA in PBMCs (Table 1). Enriched CD4+ T cell preparations contained 1.03 to 8.86 x 10^5^ copies vRNA per 10^6^ copies of *GAPDH* whereas vRNA copy numbers ranged between zero and 2.72 x 10^5^ per 10^6^ copies of *GAPDH* in CD21+ cells (Fig 4). Interestingly, the LTNP cat (187) demonstrated a reduced viral copy number in CD4+ T cells (3.07 x 10^4^) and no detectable vRNA in CD21+ cells. Cell-associated vRNA was detected in enriched CD4+ T cells derived from PBMCs in one cat (184) indicating (transient) viral transcriptional activity in circulating CD4+ T cells for this chronically infected animal. These data support the conclusion that while viral transcription is rare to undetectable in peripheral blood leukocytes during the asymptomatic phase of FIV-infection, it is readily detectable in PLNs, and multiple leukocytes subsets contribute to viral transcriptional activity.

### Viral protein detection, histology, and immunohistochemistry

**Fig 5:**
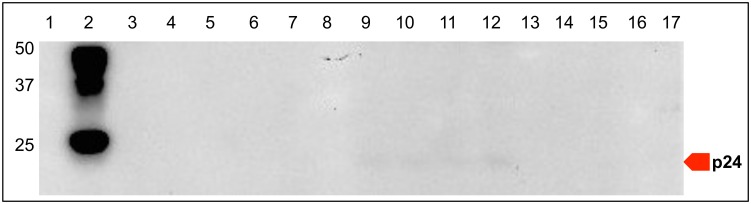
Detection FIV Gag protein by Western Blot. The p24 FIV Gag protein was detected in the positive control (*in vitro* FIV-infected MCH 5–4 cells, lane 2) and in PLN-derived leukocytes of all FIV-infected cats (lanes 9–12), but was not detected in blood-derived leukocytes (14–17). Protein size 24 KD. Lanes: (1) ladder, (2) *in vitro* FIV infected MCH 5–4 cells, (3) cat 184 PBMC 28 weeks post-FIV infection, (4) media alone, (5) FIV uninfected MCH 5–4 cells, (6) cat 185 FIV-uninfected PLN leukocytes, (7) cat 185 FIV-uninfected PBMCs, (8) ladder, (9–12) cats 165, 184, 186, 187 FIV-infected PLN leukocytes, (13) media alone, (14–17) cats 165, 184, 186, 187 FIV-infected PBMC leukocytes.

**Fig 6:**
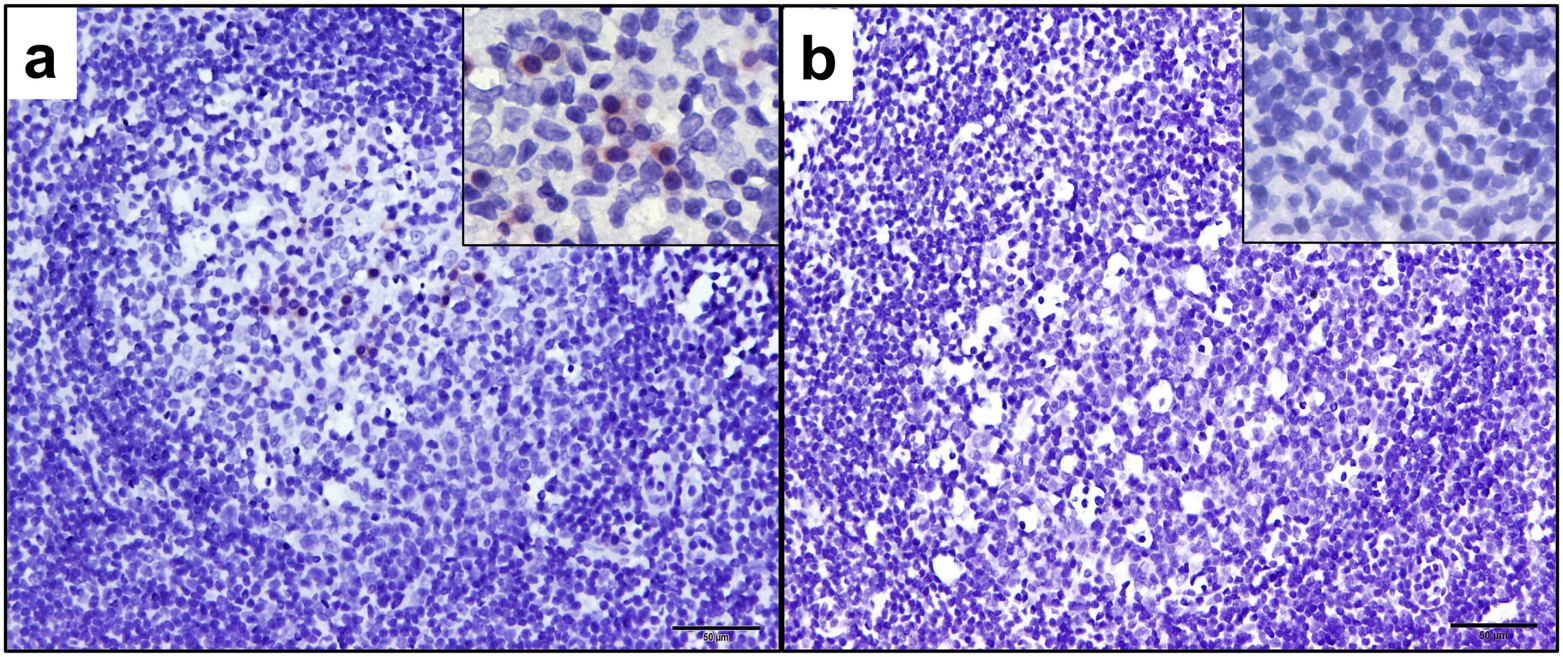
Immunohistochemistry of the PLNs for FIV antigens. Positive immunoreactivity was present in the PLNs of all FIV-infected cats. (a) Germinal center of FIV-infected cat 184 displayed at 200x using polyclonal serum from infected cat 165 (18 weeks post FIV-infection) demonstrating multiple cytoplasmic positive leukocytes within the B-cell zone. High magnification (inset at 960x magnification) demonstrates cytoplasmic immunopositivity of germinal center leukocytes. Scale bar = 50μm (b) Germinal center of FIV-uninfected control cat (185) PLN did not demonstrate immunopositivity for FIV antigens (scale bar = 50μm, inset at 960x magnification).

Unfractionated PBMCs and PLN leukocytes, and PLN tissues were examined for FIV protein production by Western blot using a FIV p24 Gag-specific monoclonal antibody and IHC. FIV p24 Gag protein was detected in PLN cell lysates from all four FIV-infected cats but was not detected in paired PBMC lysates or uninfected control cat samples (Fig 5). Immunohistochemistry analyses of PLN tissues localized FIV protein-positive cells with variable chromatic positivity within the cytoplasm of leukocytes in follicular, paracortical and medullary sinus regions of the lymph nodes of all four FIV-infected cats (Fig 6). Leukocytes lining the medullary sinuses contained the greatest concentration of FIV protein-positive cells, followed by the paracortex and follicular zones (data not shown). The uninfected negative control cat lymph node was negative for FIV protein expression as determined by IHC in all examined regions of the LN.

**Fig 7:**
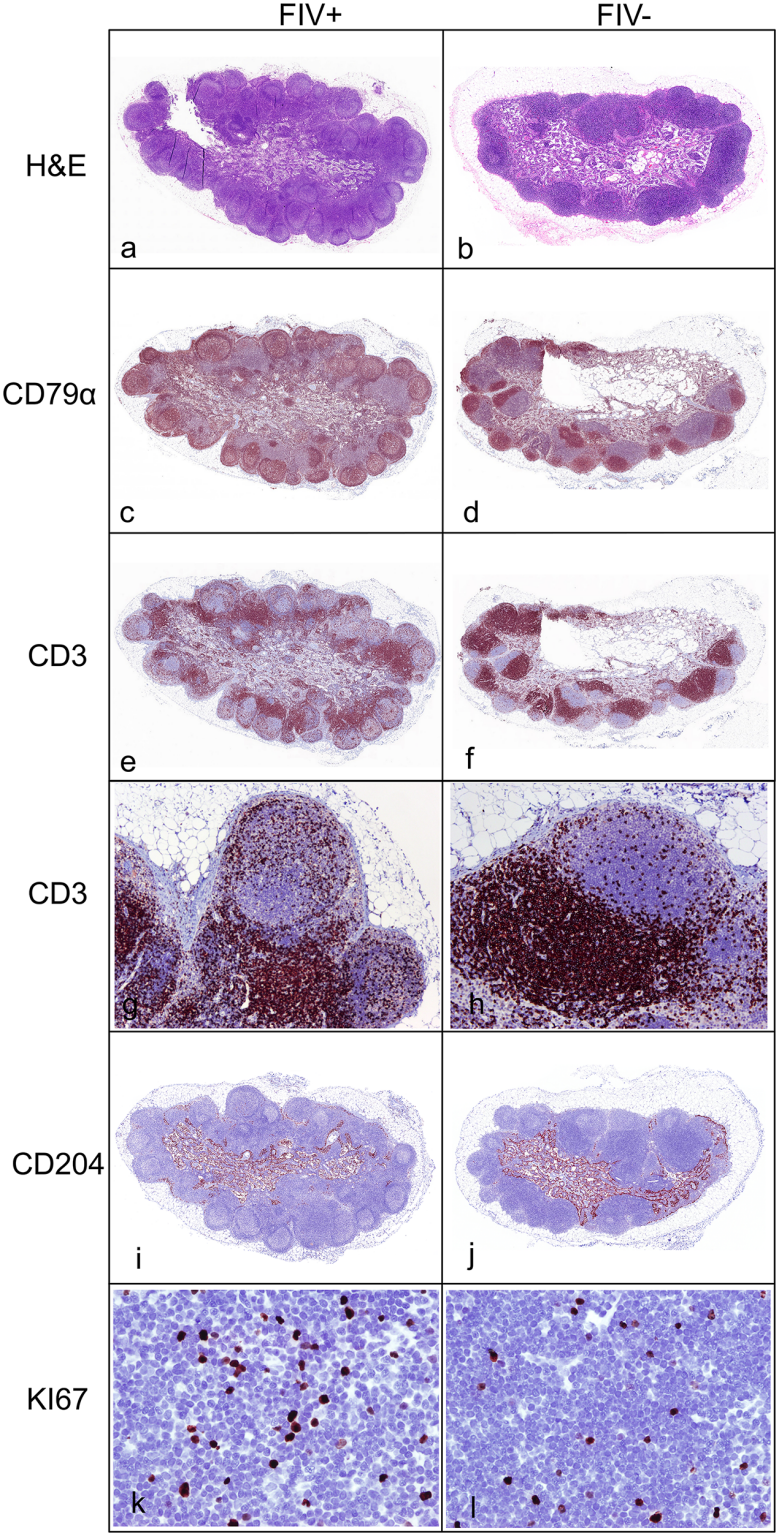
Histology and immunohistochemistry of representative FIV-infected and uninfected cats. PLNs from FIV-infected cats (a) were larger and demonstrated marked follicular hyperplasia with paracortical atrophy relative to uninfected cats (b) (Hematoxylin and eosin stains). Immunohistochemistry for CD79 demonstrated well-developed B cell germinal centers with prominent mantle zones in FIV-infected cats (c) relative to uninfected cats (d). Immunohistochemistry for CD3 demonstrated a decreased density of T cells in the paracortical zone of FIV-infected cats (e) relative to uninfected cat (f). FIV-infected cats (j) had a greater number of CD3 positive T cells in cortical B cell follicles (60x magnification) relative to uninfected cats (j) (100x magnification). There were greater numbers of proliferating Ki-67 positive cells in the paracortical T cell zones of FIV-infected cats (k) relative to uninfected cats (l) (400x magnification).

Histologically, all FIV-infected cats demonstrated marked follicular hyperplasia characterized by well-developed germinal centers with a prominent mantle cell rim and moderate paracortical atrophy (Fig 7a). Uninfected control cats showed mild follicular development and dense and discretely populated paracortical T cell zones (Fig 7b). This is consistent with the weights of the PLNs as the FIV-infected cats weighed significantly more than the uninfected nodes. Further characterization of the PLN by immunohistochemistry revealed CD79+ lymphocytes (pan-B cell marker) in boldly defined dark, light, and mantle zones of the B cell follicles of FIV-infected cats, while uninfected cats demonstrated CD79+ cells only in condensed dark and light follicular zones (Fig 7c and 7d). IHC assays highlighted the paucity of CD3+ cells (pan-T cell marker) in the paracortical zones of follicles within PLN harvested from FIV-infected cats compared to uninfected cats (Fig 7e–7h). Subjective examination of CD3 staining of lymph nodes from FIV-infected cats suggested an increased number of CD3+ T cells within the cortical lymphoid follicles (B cell zones) relative to uninfected animals (Fig 7g and 7h). Objective quantification of the frequency of CD3+ cells using image analysis software (ImageJ) confirmed that PLN from FIV-infected cats did indeed show a statistically significant greater proportion of CD3+ T cells within the B cell follicles (data not shown). The mean percentage of CD3+ T cells per PLN follicle in FIV-infected cats was 29.22 ± 8.3 and 10.69 ± 4.0 in uninfected cats. Ki67 staining was performed to investigate changes in the proliferative index of T cells in the paracortical zones of lymph nodes from FIV-infected versus uninfected cats. Image and statistical analyses indicated that FIV-infected PLNs quantitatively contained a greater replicating pool of cells in the paracortical zone (Fig 7k and 7l). In FIV-infected cats, the mean percentage of Ki67+ cells in the paracortical zone was 14.25 ± 9.63, and 3.83 ± 1.33 for uninfected cats. Immunohistochemistry for the macrophage scavenging receptor CD204 demonstrated strong positivity of macrophages lining the sinuses of the medulla, and very rarely the subcapsular sinus of both FIV-infected and uninfected cats. Subjectively, no notable differences in distribution or density of these macrophages were noted between PLNs from infected and uninfected cats (Fig 7i and 7j).

## Discussion

We have demonstrated in this cohort of cats that in the chronic asymptomatic phase of FIV-infection, there is active and productive viral replication in the PLN, despite concurrent rare to undetectable viral activity in the peripheral blood. To our knowledge, lymph node tissue viral persistence in the face of apparent peripheral blood and clinical latency has not been previously documented. Ongoing viral replication activity in lymphoid tissues provides a pathogenic mechanism by which the virus may induce chronic CD4+ T cell depletion. Direct viral killing, viral induction of apoptosis, CD8+ T cell-mediated killing and accelerated T cell turnover [19,20] are possible mechanisms that may account for CD4+ T cell depletion in FIV-infected cats.

This study details the progressive decline of peripheral CD4+ T cell numbers over a substantial amount of time (5.75 years) to extremely low levels (less than 200 CD4+ T cells/ul) in FIV-infected progressor cats. It is perhaps remarkable that these progressor animals remain clinically asymptomatic and aviremic in spite of very low numbers of peripheral CD4+ T cells. Daily observation of behavior, eating, drinking and elimination habits, serial physical examinations and serum chemistry assays, indicate an absence of overt physical illness associated with infection. In contrast to what is generally true of HIV-infected humans, researchers have noted that the CD4/CD8 ratio does not seem to directly associate with the clinical health of the cat [21].

Previous studies examining proportions of peripheral blood leukocytes of asymptomatic FIV-infected cats have also documented decreased percentages of peripheral blood CD4+ T cells; as well as a decreased CD4:CD8 lymphocyte ratio, variable percentages of CD8+ T cells, and an increased or same percentage of IgG B cells relative to uninfected cats [22,23]. Through flow cytometric methods we have demonstrated that FIV-infected progressor cats have a lower mean proportion of CD4+ T cells in both the PLNs and peripheral blood, and a higher mean proportion of CD21+ leukocytes in PLNs relative to uninfected cats. However due to the low number of subjects in our cohort, statistical significance could not be determined. A limitation of this study was the use of CD21 for phenotyping, as it is expressed on both resting memory B cells as well as dendritic cells, but is absent on activated memory B cells [24]. Future studies with additional B cell markers are warranted in order to characterize the role of B cells in FIV pathogenesis.

To determine the viral infection status of our FIV-infected cats we tested blood and PLN-derived leukocytes for proviral DNA and viral RNA loads. Results of this study revealed that mean proviral loads in unfractionated PBMCs and PLN-derived leukocytes were similar to values previously reported by Dean et al. [25] but much lower than those reported by Inoshima et al. [9]. Similar to the results of Dean and Hohdatsu et al. [26], results for this study show that total proviral loads do not vary widely between blood and lymph node compartments during the asymptomatic phase.

FIV has the capacity to infect a range of cell types including CD4+ T cells, CD8+ T cells, B cells, blood monocytes and various tissue macrophages [27]. However, the predominant reservoir during the chronic asymptomatic phase of infection has not been rigorously examined. Dean et al. found that PBMC-derived B cells contained the greatest proviral load at greater than 5 years of infection followed by CD4+ T cells and CD8+ T cells; and no significant difference in proviral load was identified between the blood and lymph node [25]. Similarly, our results show no significant difference in proviral loads between peripheral blood and PLN CD4+ T cells and CD21+ leukocyte subsets (data not shown). While quantifying the proviral load of different cell types and tissue compartments is valuable information, evaluation of viral RNA production more accurately reflects clinically relevant replication competent reservoirs.

There are very few published studies quantifying cell-associated vRNA in the chronic asymptomatic phase of FIV infection. Our results demonstrate that vRNA transcription occurs in the PLN in the face of undetectable plasma vRNA and rarely detectable cell-associated vRNA in PBMCs. Tomonaga et al. similarly observed that cell-associated viral RNA were undetectable or remained at very low copy number in PBMCs late in FIV infection [28], while Kann et al. detected plasma FIV *pol* RNA at variable copy number in naturally FIV-infected cats deemed healthy with no clinical signs [29].

In addition to vRNA, we detected FIV Gag protein by Western blot in unfractionated PLN leukocytes, but not within PBMCs obtained at the same time point. We also demonstrated that PLN leukocytes were capable of producing replication competent and infectious virus in 3 of the 4 FIV infected cats. Collectively we’ve demonstrated that the PLN contained cell-associated vRNA, viral protein, and had the capacity to produce infectious virus, all of which supports the hypothesis that there is active viral replication in the PLN during the asymptomatic phase. It is likely that the PLN is one of many lymph nodes and lymphoid tissue types with a similar viral replication profile. Our data also suggests that apparent viral latency in PBMCs and plasma aviremia do not accurately represent the overall viral replication status within the host, and that examination of tissue is required.

Results of this study demonstrated that enlargement of the PLN of all of the FIV-infected cats during the asymptomatic phase was attributable to marked follicular hyperplasia observed histologically. Follicular hyperplasia is a well-recognized response to antigen stimulation in immunosuppressive lentiviral infections resulting in expansion of antibody producing B cells [30,31]. PLNs from FIV-infected cats in our study also demonstrated notable paracortical atrophy histologically, that was supported by reduced CD4+ T cell frequencies determined by flow cytometry. Previously reported gross and histological findings of lymph nodes of FIV-infected cats are variable depending on the stage of infection and particular lymph node examined [32,33]. Lymph nodes throughout the course of FIV infection range from small to enlarged relative to control animals. Cats may have generalized lymphadenopathy or have regionally enlarged nodes draining areas of active inflammation (stomatitis, enteritis), and histologically range from follicular involution, follicular hyperplasia, to a mixed pattern [32–34]. Reports also suggest that lymph node histopathology correlates with the stage of infection with hyperplasia and mixed changes correlating with pre-AIDS stage, and involution with AIDS [33].

We noted that FIV-infected cats have an increased proportion CD3+ T cells within B cell follicles, which are likely to be T-helper follicular cells as LN germinal centers are normally populated by varying proportions of B cells, T-helper follicular cells and dendritic cells [35]. The T-helper follicular cells typically induce the differentiation of B cells to plasma cells and memory cells, and accumulate with germinal center development [36]. Interestingly, these T-helper follicular cells have been suggested as a partitioned cellular reservoir for immunodeficiency-causing lentiviruses due to the inability of cytotoxic T cells to enter the follicles [37], and are a known reservoir for HIV and SIV infections [38–40]. Also of note, previous reports revealed a high proportion of FIV-infected cells were localized to lymph node germinal centers during acute FIV infection when assayed by *in situ* hybridization of lymphoid tissues for FIV nucleic acids [4,41]. Our IHC data also reveals evidence of FIV protein production in germinal centers that is concurrent with an increase in follicular T cells, a possible reservoir for FIV infection. It should be noted that follicular dendritic cells and B cells also represent potential viral reservoirs, and our analysis does not distinguish between these different cell types. Harboring virus in follicular zones would be conceivably advantageous for the virus, as infected cells may be shielded from cytolytic destruction by virus-specific CD8+ T cells. This is the first description to our knowledge noting increased proportions of follicular T cells within germinal centers of chronic FIV-infected cats. This is a very interesting finding and future studies will be necessary to characterize this follicular T cell population for T-helper cell markers, and to determine if this follicular T cell population is productively or latently infected with FIV.

The Ki-67 protein is expressed in all stages of the active cell cycle including mitosis, and is absent in the non-proliferative G_0_ phase. It is thus commonly used as a cellular proliferation marker [42]. Given that our FIV-infected progressor cats had decreased means of CD4+ T cells in the blood and PLNs by flow cytometry, and paracortical T cell zone atrophy by histology relative to uninfected cats, we were curious to compare the proliferative index of paracortical lymph node cells. Interestingly, we found that FIV-infected lymph nodes had a greater proliferative pool in the paracortical T cell zone relative to uninfected cats (data not shown). This is interesting for multiple reasons. It suggests the paracortex of chronically infected FIV-infected cats is in a state of immunologic activation and proliferation, and should these cells be infected with FIV then they are likely replicating virus. Also, given that these cells are activated, they are prime targets for viral infection and thereby depletion. Further research into the paracortical Ki-67 cells is warranted. This IHC also indicates that the proliferative capacity of the FIV-infected cats has not been completely obliterated.

In this study we identify one of our study cats as a FIV-infected LTNP individual, which to our knowledge has not been defined specifically in FIV research. We have extrapolated this term from the HIV literature [43] and define a FIV-infected LTNP as a FIV-infected, asymptomatic cat that persistently maintains a peripheral blood absolute CD4+ T cell count indistinguishable from FIV negative cats. Our FIV negative cats had a mean peripheral blood absolute CD4+ T cell count of 2714 cells/μl and a range of 308–5630 cells/μl. The LTNP was consistently within that range and had a mean CD4+ T cell count of 3051 cells/μl for this study period. Factors associated with slow or long-term survival of FIV-infected cats have been reported in the literature but underlying mechanisms for their resistance to progression remain elusive [44]. There are multiple interesting findings from this study that characterize this LTNP cat. Most notable is that serial monitoring of peripheral blood CD4+ T cell numbers demonstrated an absolute CD4+ T cell count indistinguishable from uninfected control cats over a 5.75-year period. Relative to the progressor animals, this cat also shows a lower proviral load in unfractionated PBMCs, unfractionated PLN leukocytes, and CD4+ T cells from both blood and lymph node; but not in CD21+ cells. In addition, while vRNA was detected in PLN-derived CD4+ T cells and CD21+ B cells of progressor cats, it was only detected in PLN-derived CD4+ T cells of cat 187, and at much lower copy number relative to the progressor cats. These data support the concept that there may be a threshold of active viral replication in CD4+ T cells in lymphoid tissues that is associated with their loss. Lastly, it is interesting to note that replication competent infectious virus was reactivated from the LTNP’s PLN leukocytes, but it took a longer than the other cats. We suspect this is because, as we have demonstrated, the LTNP has a lower proviral load and therefore likely has less FIV-infected replication competent cells from which to produce infectious virus. Why we were not able to reactivate virus from PLN-derived leukocytes of FIV progressor cat 184 remains elusive.

In conclusion, our results demonstrate that there is productive viral activity in PLN tissues during the late asymptomatic phase of FIV-infection, while viral activity remains inactive in the peripheral blood. The observation that viral transcription was readily detectable in a relatively small lymphoid compartment highlights the value of using tissue, in addition to blood, as an accurate indicator of active FIV viral activity during the chronic asymptomatic phase. This study is limited in that results reflect a single time point in a single lymphoid organ in the chronic asymptomatic phase of infection. It is also limited in that the cohort of FIV-infected cats is small. Investigations into other potential tissue compartments of viral persistence and activity such as the gastrointestinal tract, mesenteric lymph node, spleen, bone marrow and brain as well as other target cell subsets are warranted to further characterize virus replication during the later stages of FIV infection.
